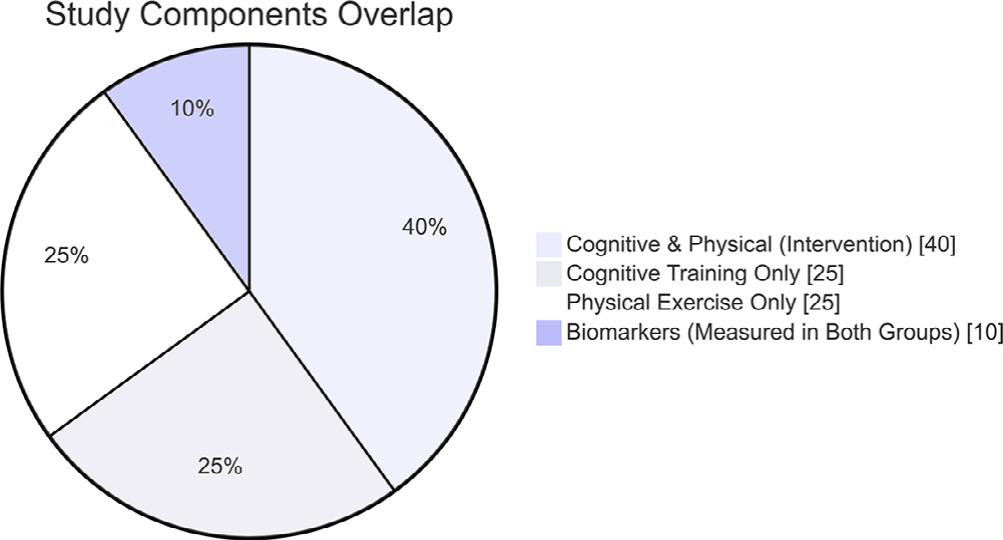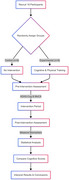# Investigating the Role of Biomarkers in Alzheimer's Disease: A Quasi‐Experimental Study

**DOI:** 10.1002/alz70856_106399

**Published:** 2026-01-07

**Authors:** MD.Hasibul Alam

**Affiliations:** ^1^ IOU International Open University, Kenifing, Banjul, Gambia

## Abstract

**Background:**

Identifying biomarkers in Alzheimer's disease (AD) is essential for early diagnosis and intervention. This study explores the impact of cognitive training combined with physical exercise on cognitive performance and neurodegeneration markers in adults diagnosed with AD.

**Method:**

Utilizing a convenience sampling approach, we will recruit eighteen participants aged 60 and above from a local community of 150 individuals. Participants will be non‐randomly assigned to either a control group (*n* = 9) or an experimental group (*n* = 9). A quasi‐experimental design and structured interview will be conducted pre‐ and post‐intervention to assess cognitive performance (using ADAS‐Cog and MoCA) and health factors. Further more the experimental group will engage in a tailored regimen designed to enhance cognitive function and target biomarkers. Additionally statistical analyses will include t‐tests to compare cognitive scores and regression analyses to investigate the relationship between biomarker levels (amyloid‐beta >300 pg/mL and tau proteins >400 pg/mL) and cognitive performance, with significance set at *p* < .05.

**Result:**

We anticipate significant improvements in cognitive performance, with higher levels of amyloid‐beta and tau proteins correlating with lower cognitive scores.

**Conclusion:**

This study aims to provide critical insights into the effectiveness of integrated cognitive and physical interventions in addressing cognitive decline in AD, emphasizing the importance of community‐based research in developing future therapeutic strategies.